# Dabigatran Reversal With Idarucizumab and In-Hospital Mortality in Intracranial Hemorrhage: A Systematic Review of Real-Life Data From Case Reports and Case Series

**DOI:** 10.3389/fneur.2021.727403

**Published:** 2021-11-24

**Authors:** Senta Frol, Dimitrios Sagris, Mišo Šabovič, George Ntaios, Janja Pretnar Oblak

**Affiliations:** ^1^Department of Vascular Neurology, Faculty of Medicine, University Clinical Centre Ljubljana, University of Ljubljana, Ljubljana, Slovenia; ^2^Department of Internal Medicine, Faculty of Medicine, School of Health Sciences, University of Thessaly, Larissa, Greece; ^3^Department of Vascular Disorders, University Clinical Centre Ljubljana, Ljubljana, Slovenia

**Keywords:** dabigatran, idarucizumab, reversal agent, intracranial hemorrhage, in-hospital mortality

## Abstract

**Background:** Intracranial hemorrhage is a severe and possibly fatal consequence of anticoagulation therapy. Idarucizumab is used in dabigatran-treated patients suffering from intracranial hemorrhage (ICH) to reverse the anticoagulant effect of dabigatran. Systematic review of real-life mortality in these patients is missing.

**Objectives:** A review of all published dabigatran-related ICH cases treated with idarucizumab was performed. We aimed to estimate in-hospital mortality rate in these patients.

**Method:** We searched PubMed and Scopus for all published cases of ICH in idarucizumab/dabigatran-treated patients until May 15, 2021. The assessed outcome was in-hospital mortality.

**Results:** We identified six eligible studies (case series) with 386 patients and 54 single case reports. In-hospital mortality rate was 11.4% in the case series and 9.7% in the case reports.

**Conclusions:** Our analysis provides clinically relevant quantitative data regarding in-hospital mortality in idarucizumab/dabigatran-treated patients with ICH, which is estimated to be 9.7–11.4%.

## Highlights

- Intracranial hemorrhage is a serious, life-threatening condition associated with anticoagulation therapy.
- Idarucizumab is a specific reversal agent of dabigatran, which achieves instantaneous reversal of anticoagulation after application.
- Idarucizumab is indicated in dabigatran-treated patients with life-threatening conditions, such as uncontrolled bleeding, or the need for urgent intervention.
- Our analysis revealed that the quantitative in-hospital mortality in idarucizumab/dabigatran-treated patients with ICH is estimated to be 9.7–11.4%.

## Introduction

Intracranial hemorrhage is a serious, life-threatening condition associated with anticoagulation therapy. Idarucizumab is a specific reversal agent of dabigatran, which achieves instantaneous reversal of anticoagulation after application seemingly without prothrombotic or other serious side effects (1, 2). The use of idarucizumab is indicated in dabigatran-treated patients with life-threatening conditions, such as uncontrolled bleeding, or the need for urgent intervention (2, 3).

Intracranial hemorrhage in dabigatran-treated patients is a rare event, with estimated yearly incidences of 0.1% for the standard dose and 0.12% for the reduced dose (4). Due to very low frequency of intracranial hemorrhage (ICH), consequently, the number of reported cases is limited. The mortality rate in dabigatran-treated patient with ICH in the REVERSal Effects of idarucizumab on Active Dabigatran (REVERSE AD) study, which included only 98 patient with ICH, was 16.4% (2). A small number of patients did not allow for firm conclusions. On the other hand, a relatively high number of reported case series and case reports were published in the following years (1, 2, 5–24).

The aim of this systematic review was to summarize all published cases of dabigatran-treated patients with ICH who were treated with idarucizumab in order to quantitatively evaluate the in-hospital mortality rate.

## Methodology

### Search Strategy and Inclusion Criteria

We searched PubMed and Scopus until May 15, 2021 for studies reporting dabigatran reversal with idarucizumab in patients with ICH using the terms: “idarucizumab” or “reversal” or “dabigatran” and “hemorrhagic stroke” or “ICH” or “intracranial bleeding.” The search was limited from January 1, 2013 to May 15, 2021, since the term “idarucizumab” was first reported in 2014. In addition, we searched the references of related letters, reviews, and editorials to identify other potentially eligible studies. We also contacted experts in the field to identify studies that have been potentially missed. To be eligible for this analysis, all studies had to provide data about the in-hospital mortality in dabigatran-treated patients with ICH after reversal with idarucizumab. This study was reported according to the PRISMA statement (25).

### Outcome and Data Extraction

The outcome assessed was in-hospital mortality. Eligible studies were assessed independently by the first two authors. Individual patient data were extracted from single case reports and case series whenever possible using prespecified forms. Quality evaluation of the included studies was conducted using a tool for the assessment of case series (26).

### Methodology and Statistical Analysis

Patient characteristics and in-hospital mortality were described based on two different prespecified approaches: individual patient data from single case reports and case series that provided individual patient data were pooled together to form a unified group of patients. Case series that provided aggregate data rather than individual patient data were reported separately.

## Results

Among 676 articles identified from the initial literature search, 22 studies were eligible and included in the analysis (1, 2, 5–24) (Supplementary Figure 1). Sixteen studies provided individual patient data of 53 patients with ICH (6–8, 10, 12, 13, 15–24), whereas six case series reported results on, overall, 394 patients (1, 2, 5, 9, 11, 14). Two studies (1, 24) included patient data that have been previously reported (7, 8). In order to avoid any duplication, in this study, we only included the data from the most recent reports (1, 24).

Quality assessment can be found in Supplementary Table 1.

Forty-four (11.4%) out of 386 patients in the case series and 4 (9.7%) out of 41 patients in the single case reports died. The characteristics of patients are summarized in Table 1.

**Table 1:** Baseline characteristics and outcomes of idarucizumab/dabigatran-treated patients with ICH.

	**Study**	**Included patients**	**Ethnicity**	**Age**	**Female**	**Traumatic ICH**	**Type of ICH**	**Death**
Case series	(5)	8	Caucasian	NA	NA	NA	NA	NA
	(9)	17	Caucasian	NA	NA	NA	NA	**5/17**
	(1)	40	Caucasian	77	12	NA	27 pts intracerebral, 11 pts subdural 2 pts SAH	**6/40**
	(11)	112		NA	NA	NA		**13/112**
	(14)	119	Chinese	NA	NA	NA	34 pts subdural, 25 pts SAH 47 pts intracerebral	**11/119**
	(2)	98	Caucasian	NA	NA	NA	39 subdural, 26 SAH, 53 intracerebral	**9/98**
	**Aggregated data from case series**	**394**	**–**	**–**	**–**	**–**		**44/386** **(11.4%)**
Case reports	Pooled single case data	**41**	2 Chinese 39 Caucasian	81 (44–94)	14/39	12/35	19pts: intracerebral hemorhage 5pts: SAH 15: subdural 2pts: mixed	**4/41** **(9.7%)**
Total	**Aggregated data from case series and case reports** [excluding (8)]	**435**		–	–	–	–	**48/427 (11.2%)**

*SAH, subarachnoid; pts, patients; RCT, randomized clinical trials; NA, not applicable; ICH, intracranial hemorrhage*.

Out of 435 patients, individual data (age, sex, traumatic/non-traumatic type of ICH, time to idarucizumab treatment, etc.) were only available for 54, which did not allow us to perform an individual patient characteristic analysis.

## Discussion

Our systematic review of data from published case reports and case series shows an estimated real-life in-hospital mortality of 9.7–11.4% in this patient population. Previous studies have reported the in-hospital mortality rate among warfarin- and direct oral anticoagulant (DOAC)-treated patients with ICH, which ranged from 25 to 33% and from 18 to 27%, respectively (27, 28). In the RE-VERSE AD study, which included dabigatran-treated patients with ICH who received idarucizumab, the described in-hospital mortality was 16.4% (2). This study, which includes data from case series and case reports, summarizes the evidence on the beneficial effect of idarucizumab in patients with ICH treated with dabigatran in order to present real-life quantitative data on in-hospital mortality.

Our review has several limitations. Heterogeneity of the cases (traumatic/non-traumatic, type of ICH: intraparenchymal/subdural hemorrhage) and missing data (age, gender, risk factors, comorbidities, timing of reversal agent application) are the main limitations of this study. Individual data were available for only 54 out of 435 patients, which could limit the assessment of how representative the cohort is. Comparison with patients with ICH who were not treated with idarucizumab would be valuable; however, there are no applicable published real-life data. Finally, we cannot exclude the presence of publication bias, particularly regarding the analysis of case reports. On the other hand, the strength of the study is consideration of all published cases.

In conclusion, our systematic review of all published cases of dabigatran-related patients with ICH treated with idarucizumab provides a real-life quantitative estimate for real-life in-hospital mortality. This estimate seems to suggest the clinically relevant efficacy and safety of dabigatran reversal in real-life treatment of patients with ICH. However, this needs to be assessed in a propensity-score matched analysis or, ideally, in a prospective study of appropriate size.

## Data Availability Statement

The original contributions presented in the study are included in the article/Supplementary Material, further inquiries can be directed to the corresponding author/s.

## Author Contributions

SF and DS: study design, data acquisition, statistical analysis and interpretation, and manuscript preparation. MŠ and GN: data acquisition, statistical analysis and interpretation, and critical revision of the manuscript. JO: study concept and design, statistical analysis and interpretation, manuscript preparation, and study supervision. All authors contributed to the article and approved the submitted version.

## Conflict of Interest

SF, MŠ, and JO reported speaker fees from Bayer, Boehringer Ingelheim, and Pfizer. GN reported speaker fees/advisory boards/research support from Abbott, Amgen, Bayer, Boehringer Ingelheim, Elpen, and Pfizer outside the submitted study. The remaining author declares that the research was conducted in the absence of any commercial or financial relationships that could be construed as a potential conflict of interest.

## Publisher's Note

All claims expressed in this article are solely those of the authors and do not necessarily represent those of their affiliated organizations, or those of the publisher, the editors and the reviewers. Any product that may be evaluated in this article, or claim that may be made by its manufacturer, is not guaranteed or endorsed by the publisher.

## Abbreviations

ICH: intracranial hemorrhage
DOAC: direct oral anticoagulant.
