# Supplementary material for: Dabigatran Reversal With Idarucizumab and In-Hospital Mortality in Intracranial Hemorrhage: A Systematic Review of Real-Life Data From Case Reports and Case Series
Source: Front Neurol. 2021 Nov 24;12:727403. doi: 10.3389/fneur.2021.727403 (PMC8653877; doi:10.3389/fneur.2021.727403)
Supplement: Supplementary file 1 [file Data_Sheet_1.docx]

Supplementary material

Identification

Records identified through literature search in PubMed and Scopus after excluding duplicates
(n = 676)

Articles excluded based on abstract: 651

- 1 paper was review
- 650 papers were not relevant

Articles assessed for eligibility
(n = 676)

Screening and eligibility

Full-text studies assessed for eligibility
(n = 25)

Articles excluded after full -text assessment: 3

Included

Studies included in quantitative synthesis
(n =22)

**Figure I :** Flow diagram of studies identified, screened and included in the analysis

| Table 1: Quality evaluation | | | | | | | | |  |
| --- | --- | --- | --- | --- | --- | --- | --- | --- | --- |
| Study | **Selection** | **Ascertainment** | | **Causality** | | | | **Reporting** | **Overall quality** |
|  | **Does the patient(s) represent(s) the whole experience of the investigator (centre) or is the selection method unclear to the extent that other patients with similar presentation may not have been reported?** | **Was the exposure adequately ascertained?** | **Was the outcome adequately ascertained?** | **Were other alternative causes that may explain the observation ruled out?** | **Was there a challenge/rechallenge phenomenon?** | **Was there a dose–response effect?** | **Was follow-up long enough for outcomes to occur?** | **Is the case(s) described with sufficient details to allow other investigators to replicate the research or to allow practitioners make inferences related to their own practice?** |  |
| Kermer, P., et al. (2020) | good | good | good | yes | no | no | yes | yes | good |
| Pollack, CV., et al (2017) | good | good | good | yes | no | no | yes | yes | good |
| Apostolaki-Hansson, T., et al (2020) | good | good | good | yes | no | no | yes | yes | good |
| Bottaro, FJ., et al. (2020) | good | good | good | yes | no | no | yes | yes | good |
| Frol, S., et al. (2018). | good | good | good | yes | no | no | yes | yes | good |
| Kermer, P., et al (2017) | good | good | good | yes | no | no | yes | yes | good |
| Küpper, C., et al. (2019) | good | good | fair | yes | no | no | yes | yes | fair |
| Phua, CS., et al. (2019) | good | good | good | yes | no | no | yes | yes | good |
| Singh, S., et al. (2020) | good | good | good | yes | no | no | yes | yes | good |
| Vene, N., et al (2019) | good | good | good | yes | no | no | yes | yes | good |
| Vosko, M., et al (2019) | good | good | good | yes | no | no | yes | yes | good |
| Yasaka, M., et al (2019) | good | good | good | yes | no | no | yes | yes | good |
| Arai, N., et al (2018) | good | good | good | yes | no | no | yes | yes | good |
| Balakumar, J., et al (2017) | good | good | good | yes | no | no | yes | yes | good |
| Edwards, G., et al (2018). | good | good | good | yes | no | no | yes | yes | good |
| Gendron, N., et al (2017) | good | good | good | yes | no | no | yes | yes | good |
| Goriacko, P., et al (2017) | good | good | good | yes | no | no | yes | yes | good |
| Quintavalla, R., et al (2018). | good | good | good | yes | no | no | yes | yes | good |
| Hieber, M., et al (2018) | good | good | good | yes | no | no | yes | yes | good |
| Sheikh-Taha, M., et al (2019) | good | good | good | yes | no | no | yes | yes | good |
| Krueger, EM, et al, (2020) | fair | fair | good | yes | no | no | yes | yes | good |
| Frol, S., et al (2020) | good | good | good | yes | no | no | yes | yes | good |
